# Commissioning and implementing a Quality Assurance program for dedicated radiation oncology MRI scanners

**DOI:** 10.1002/acm2.14185

**Published:** 2024-02-08

**Authors:** Eric Poulin, Frederic Lacroix, Louis Archambault, Jean‐David Jutras

**Affiliations:** ^1^ Département de physique de génie physique et d'optique et Centre de recherche sur le cancer de l'Université Laval Université Laval Québec Canada; ^2^ Département de radio‐oncologie et Axe Oncologie du Centre de recherche du CHU de Québec CHU de Québec‐Université Laval Québec Canada

**Keywords:** MRI in RT, QA, mri geometric distortion

## Abstract

**Purpose:**

ACR and AAPM task group's guidelines addressing commissioning for dedicated MR simulators were recently published. The goal of the current paper is to present the authors' 2‐year experience regarding the commissioning and introduction of a QA program based on these guidelines and an associated automated workflow.

**Methods:**

All mandatory commissioning tests suggested by AAPM report 284 were performed and results are reported for two MRI scanners (MAGNETOM Sola and Aera). Visual inspection, vendor clinical or service platform, third‐party software, or in‐house python‐based code were used. Automated QA and data analysis was performed via vendor, in‐house or third‐party software. QATrack+ was used for QA data logging and storage. 3D geometric distortion, B_0_ inhomogeneity, EPI, and parallel imaging performance were evaluated.

**Results:**

Contrasting with AAPM report 284 recommendations, homogeneity and RF tests were performed monthly. The QA program allowed us to detect major failures over time (shimming, gradient calibration and RF interference). Automated QA, data analysis, and logging allowed fast ACR analysis daily and monthly QA to be performed in 3 h. On the Sola, the average distortion is 1 mm for imaging radii of 250 mm or less. For radii of up to 200 mm, the maximum, average (standard deviation) distortion is 1.2  and 0.4 mm (0.3 mm). Aera values are roughly double the Sola for radii up to 200 mm. EPI geometric distortion, ghosting ratio, and long‐term stability were found to be under the maximum recommended values. Parallel imaging SNR ratio was stable and close to the theoretical value (ideal g‐factor). No major failures were detected during commissioning.

**Conclusion:**

An automated workflow and enhanced QA program allowed to automatically track machine and environmental changes over time and to detect periodic failures and errors that might otherwise have gone unnoticed. The Sola is more geometrically accurate, with a more homogenous B_0_ field than the Aera.

## 1 INTRODUCTION

Modern radiation oncology treatment, neuronavigation, and intraoperative magnetic resonance imaging (MRI) require high geometric fidelity images in combination with high spatial and contrast resolution in order to precisely identify disease extent and adjacent organs at risk (OAR). Magnetic resonance imaging (MRI) has demonstrated superior soft tissue contrast and was shown to substantially improve target and OAR segmentation accuracy and reliability.^1^, ^2^, ^3^, ^4^ In addition, it was demonstrated that MRI can reduce treatment‐related toxicities because of more accurately delineated OARs^5^, ^6^, ^7^ and identify regions of high tumor burden to facilitate dose escalation.^6^, ^8^, ^9^ However, conventional computed tomography (CT) simulation is still needed for many disease sites with target and OAR definition performed after MRI‐to‐CT image registration. This co‐registration process may introduce geometrical uncertainties in the range of ∼2 mm for the brain^10^ and pelvis,^11^ and up to 5 mm in the abdomen^12^ particularly if performed in a radiology setting, although current CT slice thicknesses and MR improvements (such as 3D distortion correction, better sequences, and so on) may improve co‐registration results. Furthermore, MR images are often used without considering the intrinsic geometric fidelity, precision and stability of the MR machine; an approach that may adversely impact dosimetric endpoints and increase the uncertainty beyond co‐registration errors. For example, a recent radiosurgery study demonstrated that geometric accuracy becomes a critical issue with small targets; for a target diameter of 3 cm, geometric distortions of 1.5 mm may impact the dose to 95% of the volume, while for targets less than 2 cm, a geometric distortion of 1 mm could significantly affect plan acceptance/quality indices.^13^

A 2011 study showed a 78% failure rate during American College of Radiology (ACR) Quality Assurance testing of MR scanners.^14^ The impact of geometric distortion in MR‐guided radiation therapy has been the object of recent studies which demonstrated that system‐related geometric distortion affects margin choice,^15^, ^16^ while the geometric distortion itself was shown to be stable over time.^16^ Overall, the impact from distortions will depend on factors such as the distance of the anatomy from the magnet isocenter, magnetic field strength, and MRI acquisition parameters and sequences, as well as MRI magnet and gradient coil properties. To address these limitations, dedicated MR simulator platforms have been recently introduced with the aim of improving the accuracy of target and OAR delineations required for radiotherapy treatment planning.^17^ Moreover, the ACR^18^ and American Association of Physicists in Medicine (AAPM) task group's^19^, ^20^, ^21^, ^22^ guidelines were recently published, addressing the specific aspects of a quality assurance program and commissioning for both cases of MR images used in conjunction with CT, as well as MR images used as a primary modality. ACR and AAPM report 284 were recently compared.^23^ To our knowledge, there is no study that combines AAPM reports and ACR testing over time and that characterizes distortion over six directions over 20 cm away from the isocenter.

The radiation oncology department has moved into a brand‐new facility and accordingly acquired two new MRIs dedicated to radiation therapy (RT). The goal of the current paper is therefore to present the authors 2 years’ experience regarding the commissioning and introduction of an extensive Quality Assurance program, with an automated workflow, for state‐of‐the‐art radiation oncology‐dedicated MRI scanners based on all recent AAPM task group reports^19^, ^20^, ^21^, ^22^ as well as ACR^18^ and Canadian Partnership for Quality Radiotherapy (CPQR) guidelines.^24^

## 2 MATERIALS AND METHODS

### 2.1 MRI systems

The department acquired a MAGNETOM Sola equipped with XQ gradients 1.5T MRI simulator dedicated to external‐beam RT planning and a Nexaris MR with MAGNETOM Aera equipped with XQ gradients 1.5T scanner for brachytherapy (both Siemens Healthineers, Erlangen, Germany). The Sola, launched in 2018, is the most recent MRI system compared to the Aera, which was the previous system. The Sola was installed with the *Syngo* MR XA20 software platform (later upgraded to XA31 on November 2021) while the Aera was configured with the *Syngo* VE11E software platform on March 2021 (later upgraded to XA30 on September 2021). The Sola field of view (FOV) is 50×50×50 cm^3^ while the Aera FOV is slightly shorter in the longitudinal dimension and covers 50×50×45 cm^3^. Vendor specific phantoms includes a 25 cm sphere for B_0_ homogeneity, and various cylindrical phantoms and custom‐fitting foam mats for antenna channel uniformity testing.

### 2.2 Commissioning and QA

The AAPM as well as the CPQR have drafted recommendations for the establishment of Quality Assurance (QA) programs^19^, ^20^, ^21^, ^24^ and commissioning^19^, ^20^, ^22^ for dedicated MRI RT systems. The goal of this QA program is to insure the best possible performance of RT MRI scanners, while optimizing or minimizing the QA time requirements. A significant effort was made to automate the data analysis as much as possible through the use of vendor, in‐house or third‐party software. QA data logging and storage was performed using QATrack+, an open source database for managing QA data.^29^ Every test contains one or more measurement results that are uploaded in QAtrack+ and monitored over time. ACR results are also automatically imported in QATrack+ through a Python script using libraries such as Openpyxl.

All mandatory commissioning tests suggested by the AAPM report 284^19^ were performed. QA tests and associated tolerances are defined to ensure the highest possible geometric accuracy, image integrity and stability of RT MRI images. In the supporting material, Tables 1‐4 summarizes the QA program established at our department. It was decided to perform monthly 3D distortion, vendor homogeneity, and RF tests, which constitutes a higher frequency than recommended in AAPM report 284. An in‐house version of the ultra‐fast imaging Echo‐planar imaging (EPI) test described in AAPM report 100 was also implemented. A variety of analysis methods are used: visual inspection, in‐house tools provided by the vendor clinical or service platform, third party software (e.g., AutoQAplus from QA Benchmark, Maryland, USA, and GRADE QA software, Spectronics Medical, Helsingborg, Sweden) or in‐house python‐based code. In the QA program established at the department, vendor service tests and platform were used since they correspond to AAPM tests as described in a white paper^25^ and the prescribed methodology was followed; ACR testing is also shown. The ACR analysis is performed using AutoQAplus v1.7.4.0 (QA Benchmark, Maryland, USA) and the software allows for the automatic analysis of all the ACR tests except the high‐contrast spatial resolution, while the low‐contrast object detectability can be performed automatically or manually; it also allows automatic export of the results. The software was validated by comparing its results to a manual analysis over 10 different scans selected randomly over a period of 3 months using the standard ACR head coil, but also using a combination of body and spine coils. The same validation was performed for one scan on images originating from various machines such as GE Healthcare (1.5T Artist and Explorer), Philips (1.5 T Ambition), Siemens (3T vida fit), Viewray MR‐Linac, and Elekta Unity.

Gradient nonlinearities can be the largest‐contributing source of geometric distortions.^26^ It has been demonstrated that the residual distortions once vendor‐supplied 3D correction factors are applied can be greater than 1 mm at 10 cm from the isocenter.^19^ Therefore, geometric distortions were characterized using the GRADE QA phantom (Spectronic Medical SE, Karbingatan, Sweden). The GRADE QA phantom was recently described^27^ and is of large diameter (47.9 cm lateral, 38.5 cm height, and 50 cm longitudinal). The GRADE QA phantom thus enables a characterization of the full scanner FOV.^27^ A 3D Fast low angle shot (FLASH) sequence was used to image the GRADE QA phantom as recommended.^27^ The sequence parameters (*FLASH3D_geo*) are provided in Table 5 of the supporting material. As suggested in AAPM report 284,^19^ the same scan was repeated using opposite readout gradient polarities along each axis: LeftRight (LR)/RightLeft (RL), AnteroPosterior (AP)/PosteroAnterior (PA), and SuperoInferior (SI)/InferoSuperior (IS). The phantom was also used to evaluate and optimize the geometric distortion in clinical sequences, for example, in 3D Magnetization‐prepared Rapid Gradient Echo (MPRAGE) and 3D turbo spin echo (SPACE) pulse sequences used for brain stereotactic radiosurgery planning respectively^28^ (FOV: 240 × 240 × 176 and 250 × 250 × 176 mm^3^, Acquisition matrix: 384 × 384 and 320 × 320, axial slice thickness 1 mm for both, TI/TR_shot_/TR/TE/α= 845/1670/8.16/3.31 ms/10° and TR/TE/Turbo factor/α = 600/19 ms/42/Variable T1, iPAT = 2 for both, Bandwidth = 160 and 558 Hz/pixel).

### 2.3 In‐house experience

For five of the quality assurance tests recommended by the AAPM reports, or the CPQR, no dedicated commercial software exists yet; therefore, in‐house python programs were developed to perform an automatic analysis. The analysis of those five tests is described below:

#### 2.3.1 EPI geometric distortions

For the EPI geometric distortion check (AAPM Report 100^20^), an EPI pulse sequence is employed and shown in Table 5 of the supporting material. An additional turbo spin echo (TSE) sequence with the same FOV, slice positions and acquisition/reconstruction matrix size (Table 5 of supporting material) is used as a geometric reference to measure the EPI distortion in both frequency‐encode and phase‐encode directions. The ITK Canny Edge Detection Filter was used to automatically detect the edges, after optimizing the parameters based on a manual edge detection, of both the TSE and the EPI image as shown in Figure 4 of the Results section. From the edge masks, the distances in the central row and column of the images are automatically subtracted, yielding a relative measure of distortion in the frequency and phase directions to an accuracy in the order of the image resolution (1 mm).

#### 2.3.2 EPI ghosting ratio

The EPI average ghosting ratio is calculated using Equation 1 within the central uniform slice of the ACR phantom. The EPI sequence used is the same as for the EPI distortion and is listed in Table 5 of the supporting material.

(1) $$\%\mathrm{GR}=\left|\frac{\left(S_{L}+S_{R}\right)-\left(S_{T}+S_{B}\right)}{2S_{C}}\right|\times 100\%$$

Here, S_L_, S_R_, S_T_, S_B_, and S_C_ are the average signal in the left‐side, right‐side, top, bottom and central Regions of interest (ROI) locations. The ROIs, as shown in Figure 6 of AAPM report 100,^20^ are initially drawn manually and then saved as masks which can be re‐used indefinitely to improve the consistency or reproducibility over time. A threshold of 3% was selected as the acceptable upper limit for ghosting.

#### 2.3.3 EPI long‐term stability

The EPI long‐term stability check was based on two previous publications, the Glover stability QA protocol (GSQAP)^30^ and the ROI analysis of Weisskoff.^31^ Both analyses were performed on the same uniform ACR phantom slice of the *EPI_stability* sequence with parameters listed in Table 5 of the supporting materials.

#### 2.3.4 B_0_‐inhomogeneity

Although the B_0_‐homogeneity phantom shim check on the vendor service platform (customer platform as of Syngo MR XA31 for Sola) is run on a monthly basis (Table 2 of supporting material), a common B_0_‐inhomogeneity mapping technique described in both the AAPM report 284^19^ and Report 100^20^ was also implemented as an independent verification. The Body‐coil was used as the receiver to avoid phase inconsistencies, which might be present in multichannel head coils. The *unwrap_phase* algorithm as part of the *skimage.restoration* Python toolkit was found to be sufficiently robust to perform 3D phase unwrapping on the 25 cm vendor‐supplied spherical phantom images of a double‐echo spoiled gradient echo sequence (*FLASH3D_B_0_*) listed in Table 5 of the supporting materials. The following equation is used to quantify the final field inhomogeneity in parts per million (ppm):

(2) $$\Delta B_{0}\left[ppm\right]=\frac{\Delta \phi \left[rad\right]\left(42.576\frac{MHz}{T}\right)}{\gamma \left[\frac{rad}{Ts}\right]\Delta TE\left[s\right]f_{0}\left[MHz\right]}\times 10^{6}ppm$$

Here, $\Delta \phi =\phi_{2}-\phi_{1}$ is the phase difference after unwrapping the phase $\phi_{1}$, $\phi_{2}$ corresponding to each echo time (TE) TE_1_, TE_2_ with difference $\Delta TE\;=\;TE_{2}-TE_{1}$, $f_{0}$ is the Larmor frequency (in MHz) of the scanner, and γ is the proton gyromagnetic ratio. B_0_ maps were used in combination with the installation shim report, using an accurate 24‐plane plot with 20 angles,^32^ to extract maximum diameter spherical volumes (DSV) for different RT applications and were compared to Gach et al.^33^

#### 2.3.5 Parallel imaging

The SNR verification of parallel imaging was performed using the image difference method (described in AAPM TG‐118^21^), via two 3D MPRAGE measures without acceleration (*R* = 1) and two with Generalized Autocalibrating Partial Parallel Acquisition (GRAPPA) acceleration (*R* = 2), including 48 integrated k‐space lines. The signal‐to‐noise (SNR) ratios of *R* = 1 to *R* = 2 were then measured in five square ROIs inside the uniform slice of the ACR phantom image (as shown in Figure 1 of the supporting material) to assess SNR stability over time when using parallel imaging. The 3D MPRAGE pulse sequence for brain stereotactic radiosurgery planning with integrated Parallel Acquisition Technique (iPAT) = 2 was used.

The GRAPPA^34^ parallel imaging method allows the possibility to acquire the reference lines in an integrated or separate method. The separated method uses a gradient echo (GRE) sequence and allows the user to choose the number of reference lines in both phase directions (in plane and slice). The impact of the reference line acquisition method on SNR and time was evaluated using the methodology described in the parallel imaging SNR stability test with the ACR phantom. The MPRAGE sequence was used and the acquisition time was recorded for each acquisition. For the integrated method, the number of lines was varied from 24 to 96 while for the GRE method the following configurations were tested: 64 in‐plane lines and 24 slice lines (64‐24) as well as 96 in‐plane lines and 48 slice lines (96‐48).

## 3 RESULTS

**FIGURE 1:**
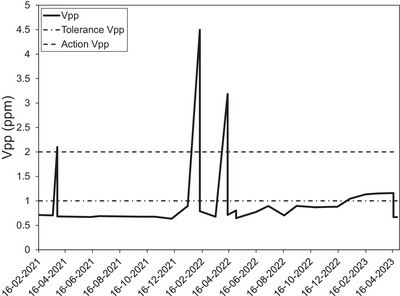
Volume peak‐to‐peak B0 inhomogeneity (V_pp_ in ppm) using the phantom shim check over a period of 24 months.

The complete AAPM TG 284, TG 100, and CPQR tests were performed as part of commissioning and no failures were detected on both the Aera and Sola. However, vendor provided SNR references were inaccurate for the Body‐30 receiver coil and as a consequence the vendor QA test failed systematically; the errors were detected and could be resolved in Syngo MR XA version 31. The vendor service platform allows for automatic QA processes (some QA processes are available on the customer platform with Sola (1.5T) and Vida (3.0T) scanners on syngo MR XA31 and later versions). These tests take less than an hour to run and they meet major AAPM TG 284 and TG 100 recommendations; vendor‐specific tests used in the QA program are identified in supplementary Tables 1 to 4. Phantom shim check and gradient sensitivity check were independently verified. The service or customer QA platform allowed to perform semi‐automatic coil tests based on the TG 284 recommendations. The time needed for testing depends on the coil itself (and is mainly dependant on the number of channels and length), and can range from 5 min to more than 20 min (e.g., for the spine‐32 array or Body‐30). All monthly tests were performed in approximately 3 h. Low‐specific absorption rate (SAR) radio frequency (RF) pulses (default setting in some sequences) were found to cause the ACR slice selection thickness test to fail with a value of 6.1 ± 0.2 mm, which was outside the tolerance of 5.0 ± 0.7 mm. The shim and gradient sensitivity had to be re‐tuned during the first 9 months of operation for both machines, as well as the Body Coil tuning for the Sola. The shim tuning was needed within the first 2 months post‐commissioning and ramping up of the magnet. Figure 1 shows the B0 inhomogeneity [Volume peak‐to‐peak (V_pp_) result] of the Sola system over a period of 24 months using the phantom shim check. After the first two months, the cause of two values being measured outside of the action threshold were due to a metallic construction bin placed by error by a construction contractor outside adjacent to the MRI. Spike and RF noise check were found to be out of tolerance for the Aera system 12 months post ramp‐up; the cable of the in‐room surveillance camera was identified as the source of the noise. The daily ACR geometric accuracy test was found to be out of tolerance 24 months post ramp‐up, with a value of 192.3 mm, outside the 2 mm tolerance. The problem was detected post‐gradient tune‐up and the vendor identified the calibration sphere phantom as the source; the geometric accuracy was back inside the tolerance after changing the sphere and performing tune‐up again. The AutoQAplus software was shown to be accurate and validated using manual measurements, only the automatic low contrast detectability test results were found to differ significantly (*p* < 0.001; paired Student‐*t* test) from the manual analysis results with an average 34 ± 2 spokes for the automatic test compared to 31 ± 3 for the manual test. Diameter (190.4 ± 0.2 vs. 190.6 ± 0.4), percent intensity uniformity (93 ± 2 vs. 94 ± 3), ghosting ratio (0.006 ± 0.003 vs. 0.005 ± 0.003), slice thickness (5.1 ± 0.2 vs. 5.3 ± 0.3), and slice offset (−0.1 ± 1.1 vs. −0.2 ± 1.2) were not significantly different between automatic and manual measurements. On average, manual measurements were shown to have a significantly higher standard deviation than automatic measurements. The results obtained with AutoQAplus for other MRI models or other MRI / MR‐Linac vendors were similar.

### 3.1 Distortion characterization

**FIGURE 2:**
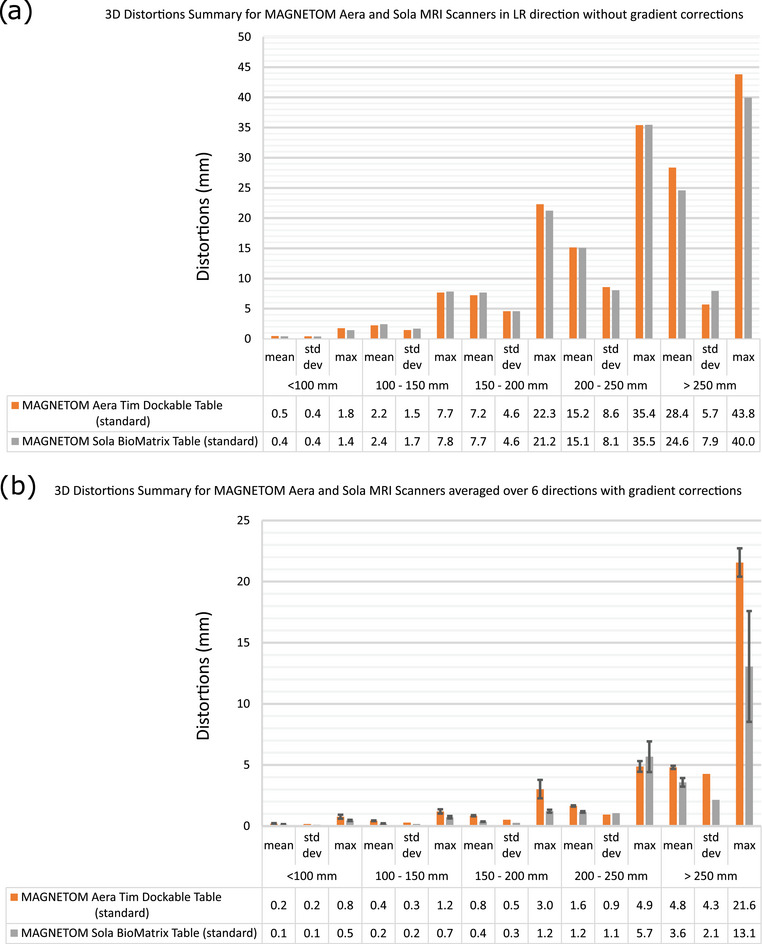
3D distortion mean, standard deviation and maximum values across spherical volumes of various radii from the imaging isocenter for the Sola and Aera scanners for (a) the Left‐Right (LR) phase encode direction without gradient corrections and (b) averaged over six directions with gradient correction on; the error bars are the standard deviations measured across the six different directions.

Figure 2 presents the mean, standard deviation, and maximum 3D distortion values for different radii spheres centered around the imaging isocenter for the Sola and Aera scanners. It first illustrates the large effect of turning the 3D gradient corrections “On” when comparing (a) 3D distortion in LR direction without gradient correction and (b) 3D distortion averaged over six directions with gradient correction “On.” The main effect is a halving, or more, of the mean and maximum residual distortion across the scanner FOV which illustrates that applying the vendor‐supplied gradient correction mitigates, to a large extent, the residual 3D distortion. On the Sola, the average distortion is on the order of 1 mm for imaging radii of 250 mm or less. For radii of up to 200 mm, the maximum, average (standard deviation) distortion is 1.2  and 0.4 mm (0.3 mm). Mean, standard deviation, and maximum distortion values on the Aera standard table are roughly double the Sola values for radii up to 200 mm and there is therefore a trend for lower average residual distortion on the Sola. Supplementary Figure 2 illustrates the distortion vector magnitude plots as a function of the distance from the MRI isocenter for the Sola and Aera platforms. Both Sola and Aera residual 3D distortions were stable over 2 years. MPRAGE and SPACE brain stereotactic radiosurgery sequences maximum distortion were 1 and 0.8 mm, respectively, while the mean distortion was 0.4 mm over the FOV of interest for both sequences.

### 3.2 B_0_ inhomogeneity

**FIGURE 3:**
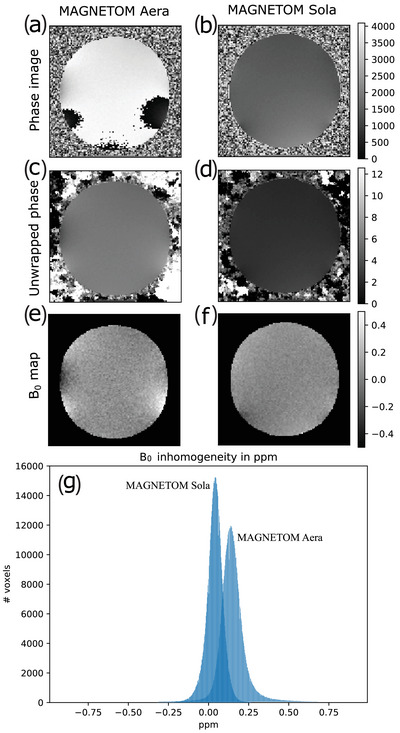
B_0_ homogeneity test showing (a) wrapped phase image, (c) unwrapped phase image, (e) B_0_ inhomogeneity map in ppm for the Aera system, while (b), (d), and (f) are equivalent for the Sola system. Sub‐figure (g) is showing the histogram of the inhomogeneity superposed for both systems.

Results of the B_0_ inhomogeneity mapping in the 25 cm sphere for the Aera and the Sola 1.5T scanners are compared in Figure 3 in a sagittal slice. The mean of the unwrapped phase (in c and d) can be close to any multiple of 2π, which explains the different intensities. The maximum DSV calculated for ultrafast applications was 30.4 cm for the Sola and 26.8 cm for the Aera while for whole body RT the values are 39.1  and 34.5 cm, respectively.

### 3.3 Advanced imaging

**FIGURE 4:**
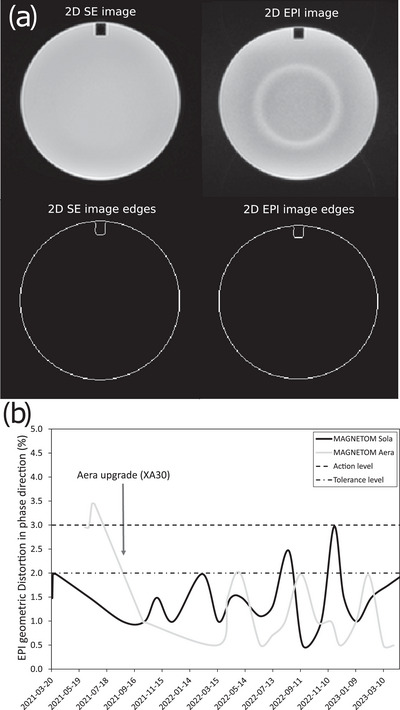
EPI distortion test showing (a) the mask identified by the authors’ analysis, (b) EPI distortion data accumulated over 24 months for both Sola and Aera scanners.

**FIGURE 5:**
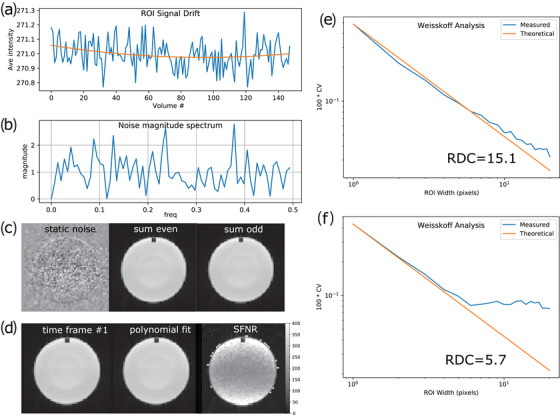
EPI stability test showing (a) the signal drift over time (8 min 30 s), (b) the noise magnitude spectrum from the accumulated data, (c) the static noise as well as the even and odd data sum together, (d) image of the first time‐frame in combination with the corresponding polynomial fit of that data and the SFNR result. Weisskoff analysis of an (e) acceptable and (f) inacceptable results.

Figure 4 shows some phantom images and edge detection results from the automated EPI geometric distortion measurements. The distortion in the phase encoding direction in (b) is shown for both scanners over a period of 24 months. The range of EPI distortions was between 0 and 2 mm for the Sola, while for the Aera it was between 6 and 8 mm before the upgrade to XA30 as well as the ACR phantom refilling (to eliminate air bubbles) and in the same range as the Sola after the upgrade. The average EPI ghosting ratio was (mean ± σ) 1.4 ± 0.6% and 1.0 ± 0.3% for the Sola and Aera scanners respectively. Example results of the EPI long‐term stability test are shown in Figure 5a‐d for the Glover Stability QA protocol and Figure 5e,f for the Weisskoff analysis. The mean percent drift was (mean ± σ) 0.10 ± 0.09% and 0.09 ± 0.05%, while the mean percent fluctuation was (mean ± σ) 0.038 ± 0.005% and 0.033 ± 0.002% for the Sola and Aera scanners, respectively. In addition, the mean RDC was (mean ± σ) 11.8 ± 1.9 and 14.7 ± 0.8 for the Sola and Aera scanner, respectively.

**FIGURE 6:**
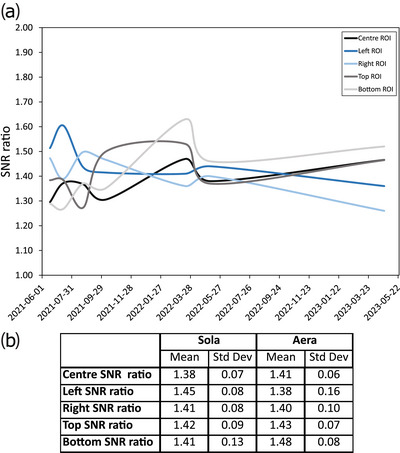
Parallel imaging stability test showing (a) a plot of SNR for the five identified regions of Figure 2 over a period of approximately 22 months and (b) the table showing the mean and standard deviation over the five different regions.

**FIGURE 7:**
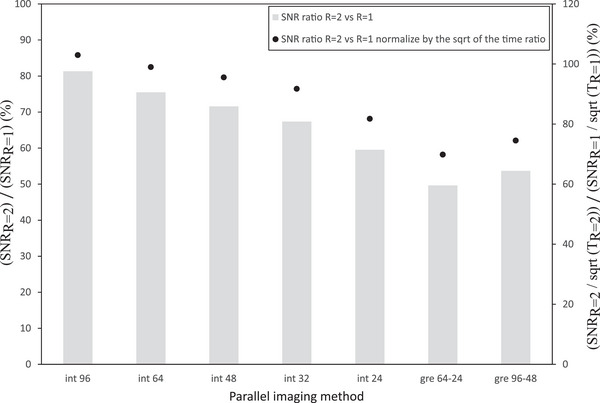
Impact of the number of reference lines and calibration method on the SNR (central ROI of Sup. Figure 1) with GRAPPA and SNR efficiency, defined as the ratio of SNR to square‐root of scan time.

The parallel imaging SNR ratio stability is plotted in Figure 6 over a period of 22 months for the five ROI locations shown in supplementary Figure 1. The average SNR ratio over the five locations is close to 1.41 (√2) for both systems. Figure 7 shows the impact of the number of reference lines and calibration method on the SNR with GRAPPA, including the imaging time to incorporate a metric of efficiency. The results show a significant improvement in SNR and efficiency with the integrated calibration in comparison with the gradient echo separated calibration. There was a 17% decrease in SNR between 24 integrated lines and 64‐24 gradient echo configuration. The SNR improved with the number of reference lines with approximately a 37% difference between 96 and 24 integrated lines.

## 4 DISCUSSION

The complete end‐to‐end testing required by the AAPM task group reports^19^, ^20^, ^21^ were performed without major issues. Coil QA errors (Body‐30) demonstrated the importance of testing coils as part of the acceptance testing. The vendor service and clinical platforms^25^ allowed us to cover most of the AAPM recommended tests in a time‐efficient manner. The MRI physicist in charge of QA should identify vendor‐supplied tests that satisfy AAPM recommendations. Furthermore, the vendor service platform makes it straightforward for the MRI physicist to directly address any failing test or scanner issue. Special attention should be given to shimming, particularly within the first 2 months of the magnet ramp‐up. Contrary to the recommendations of AAPM report 284, the homogeneity as well as RF noise and spike test were verified monthly. This allowed us to detect a major B_0_ homogeneity problem when a metallic construction container was placed outdoors next to the MRI. An environmental change contributed to spurious noise coming from the camera cable; RF noise and spike check can be used to validate that third‐party items will not produce RF interference. From the authors’ experience, B_0_ homogeneity can drift slowly or rapidly and RF interference can be introduced by changes in the MRI room or in its vicinity; the authors therefore recommend homogeneity and RF tests to be performed monthly. The gradient sensitivity was also an important parameter to verify, as both machines needed to have their laser‐to‐isocentre distance re‐adjusted (i.e., the programmed table motion that automatically brings the patient or phantom to isocentre after zeroing on the scanner's laser, which depends on the gradient sensitivity).

The vendor gradient sensitivity test in combination with the 25 cm sphere can be used to evaluate the geometric accuracy over a FOV of 25 cm as stated in AAPM report 284^19^ and this resolves the FOV size issue stated in the study of Buatti et al.^23^ In addition to the vendor geometric test, daily independent ACR phantom testing allowed to detect a gradient tune‐up error and correct it; this would have been missed with only the gradient sensitivity check provided by the vendor. For radiotherapy purposes, low‐SAR RF pulses should be avoided in order to prevent thicker slices than expected (potentially biasing the MR‐CT registration through‐slice accuracy).

One limitation of this study is that not all vendor specific tests could be validated (e.g., eddy current test), due to the complexity of implementing independent tests. It would be of interest for the MR community to collaborate on implementing independent “gold standard” tests to validate these vendor specific tests.

The AutoQAplus software was shown to be accurate to automatically analyze ACR tests, however due to the differences between manual and automatic measurements the low contrast detectability test was performed manually. Results demonstrated that the software is also accurate with other MRI vendors, such as GE Healthcare and Philips, and MRI‐Linac vendors (Elekta and Viewray). Although AutoQAplus can be used for trend analysis, all the data were uploaded in QATrack+ for trend analysis and follow‐up as per our center's QA program.

The geometric distortion characterization showed that the vendor‐implemented 3D correction algorithm mitigates, to a large extent, the geometric distortions on a sizable portion of the FOV in all 6 readout directions (at least up to a 20 cm radius). The 3D‐corrected average distortion is less than 1 mm within a 10 cm radius and less than 2 mm within a 25 cm radius around the isocenter on both the Aera (standard table) and Sola scanners. These values were found to be within the recommendations of report 284^19^ and the measured distortion showed a clear downward trend on the Sola compared to the Aera. These values can be used as an estimate for the magnitude of system‐related distortions on both the Sola and Aera platforms. The 3D distortion was found to be stable over 2 years in agreement with Lu et al.^16^ and should be performed annually and after major upgrades. 3D distortion phantoms can be used to estimate and optimize the distortion of clinical sequences, for example, the brain stereotactic radiosurgery sequence was optimized to a maximum distortion of 1 mm. Using a different distortion evaluation method may impact the results depending on the phantom field of view, number of data points and analysis method; however the repeatability and set‐up sensitivity of the current method was validated in a previous study.^27^

The superior B_0_ homogeneity of the Sola in comparison to the Aera is clearly visible in Figure 3e and f and is confirmed by the histogram of the entire FOV in g). There is a significant difference between the histogram centers of approximately 0.1 ppm. In order to ensure a fair comparison, the same standard shimming routine was used on both systems. Therefore, the narrower full width at half maximum of the ∆B_0_ histogram for the Sola scanner is due to the superior intrinsic homogeneity of this magnet. Using the Gach et al. study,^33^ the Sola homogeneity was found to be within the top 5%, particularly for ultrafast imaging, while the Aera is within the 25−75% interquartile (depending on the application) of the machines evaluated in this study.

The EPI distortions on the Aera scanner were reduced from 3.4% following the system installation with VE11E software to below 2% after a system software upgrade (to version XA30), which enabled using the exact same EPI sequence on the Aera as on the Sola scanner for comparison (version XA20). The ACR phantom needs to be periodically refilled, approximately once per year, in order to eliminate air bubbles within the phantom (especially in the uniform slice of interest), as air can significantly increase the geometrical distortions locally, which could be responsible in part for the improvement seen after the upgrade. High distortions locally near the air bubbles indicate the need for phantom refills. The ghosting ratio test is sensitive to the receiver coil arrangement and the image intensity correction filter. In fact, the intensity correction filter was found to be responsible for increasing the noise floor around the image, consequently exaggerating the ghosting ratio. Therefore, we deemed that either a moderate or no intensity correction is preferable for this QA test.

As demonstrated in Figure 5a the mean signal drift is minor on both scanners tested in the current study and we did not observe a consistent tendency toward a positive drift as had been claimed previously.^30^ Rather, the drift was occasionally positive and negative. The mean drifts reported in the current study for the Sola and Aera are significantly lower than those reported by Glover et al. (0.3% at the lowest),^30^ suggesting an improvement in scanner hardware stability in the last twenty years. The difference in average RDC is attributable to different software versions (before the Aera was upgraded to XA30). Glover et al. demonstrated that a low RDC is often associated with a non‐stationary and irregular time course of the residuals (after polynomial fit of the 150 dynamics).

In the parallel imaging stability test, an average SNR ratio over the five locations was found to be close to 1.41 ( = √2), which is what one would expect in the case of a geometry factor (g factor) of 1. Normally, the geometry factor is greater than unity, but if a regularization is employed in the parallel imaging reconstruction, values below unity are possible.^35^, ^36^ However, a desirable amount of regularization should approach a g factor of ∼1, which can be assumed when making SNR predictions on 3D MRI protocols with varying factors of acceleration.^37^ In GRAPPA parallel imaging, the calibration method was shown to directly impact the SNR. In fact, the integrated method was shown to be significantly better in terms of SNR with no significant difference in scan time compared to the GRE method. The SNR increases with the number of reference lines. A previous study recommended a minimum of 32 integrated lines to be used with GRAPPA.^34^ The current study is in agreement with Blaimer et al.^34^ and further suggests that 48 integrated lines better preserves SNR efficiency since the acquisition time was minimally impacted. High‐resolution imaging is needed for radiotherapy planning, making it more difficult to preserve sufficient SNR in an acceptable scan time; therefore we recommend using 48 integrated lines with GRAPPA for these sequences. Using a different coil combination or a low channel number flexible coil with a thermoplastic mask may affect the results, as the g factor noise will be different.

## 5 CONCLUSION

No major issues were detected during acceptance and commissioning. An automated workflow and enhanced QA program, monitoring vendor and in‐house tests, allowed to automatically track machine and environmental changes over time as well as detect periodic failures and errors that might otherwise have gone unnoticed. In fact, periodic problems, such as B0 homogeneity and RF interference, were detected within the first two years and this demonstrated the importance of monthly QA. The Siemens service platform and automated in‐house workflow was useful and time‐saving in permitting medical physicists to perform most of the QA program in a semi‐automatic or automatic fashion and in a clinically‐acceptable time; this should be investigated by the MRI physicist doing QA on other vendor scanners. The Sola system is found to be more geometrically accurate, with a more homogenous B_0_ field making it better suited than the Aera for external‐beam RT planning. GRAPPA parallel imaging reference lines and calibration methods were optimized in order to obtain a sufficient SNR in a clinically acceptable scan time for radiation therapy simulation.

## CONFLICT OF INTEREST STATEMENT

The authors declare no conflicts of interest.

## Supporting information

Supporting Information
