# Supplementary material for: Commissioning and implementing a Quality Assurance program for dedicated radiation oncology MRI scanners
Source: J Appl Clin Med Phys. 2024 Feb 8;25(3):e14185. doi: 10.1002/acm2.14185 (PMC10929993; doi:10.1002/acm2.14185)
Supplement: Supplementary file 1 — Supporting Information [file ACM2-25-e14185-s001.pdf]

SUPPORTING MATERIAL

Supplementary Table 1 : MRI Daily Quality Assurance (QA) tests performed as part of a Radiation Therapy QA program.

**Daily QA**		
**Test**	**Analysis Method**	**Guidelines recommandations (frequency) [differences]**
Safety and emergency (cameras, intercom, emergency buttons, O_2 sensor)	Visual	TG 284 (daily)
Mechanical QA (table, antenna and connector integrity, temperature, helium, water level)	Visual	CPQR (daily)
American College of Radiology (ACR) phantom with alternate daily coil setup (Monday-Friday)	QA Benchmark software	CPQR (daily)<br>Basic geometry in TG 284 (daily)
Image origin correspondence with LAP lasers (Sagittal localizer sequence on ACR)	Siemens clinical platform	TG 284 (daily)
Transmit gain setting (T1 Spin Echo sequence on ACR)	Siemens clinical platform	TG 284 (daily)
SNR stability (T1 Spin Echo sequence on ACR)	Siemens clinical platform	TG 284 (daily)

1. These QA tests are available on the Siemens customer platform with MAGNETOM Sola and Vida scanners on syngo XA31 software and later versions, only. Otherwise, the tests are available on the Siemens service platform.

Supplementary Table 2 : MRI Monthly Quality Assurance tests performed as part of a Radiation Therapy QA program.

**Monthly QA**		
**Test**	**Analysis Method**	**Guidelines recommandations (frequency) [differences]**
Coil QA (for all regularly-used coils e.g. Body-18, Body-30, Head&Neck-20, UltraFlexLarge-18, Spine-32)	Siemens service platform	TG 284 (monthly for flexible coil)
RF noise check	Siemens service platform	AAPM Report 100 (no recommendation)
RF spike check	Siemens service platform	AAPM Report 100 (no recommendation)
B_0 homogeneity phantom shim check	Siemens service platform / Siemens customer platform[1]	TG 284 (annual)
Helium level and cooling water flow rate	Siemens service platform	TG 284 (monthly)
RF artifact check (based on the ACR image data of the month)	Visual	TG 284 (monthly)
3D geometric distortion QA with gradient correction using 3D GRADE phantom (gre_ax_LR only)	Spectronics software	TG 284 (annual)
EPI average ghosting ratio check (on ACR phantom)	In-house python code	ACR (annual)
EPI geometric distortion check (on ACR phantom)	In-house python code	AAPM Report 100 (no recommendation)
EPI long-term stability check (on ACR phantom)	In-house python code	AAPM Report 100 (no recommendation)

1. These QA tests are available on the Siemens customer platform with MAGNETOM Sola and Vida scanners on syngo XA31 software and later versions, only. Otherwise, the tests are available on the Siemens service platform.

Supplementary Table 3 : MRI Semi-Annual Quality Assurance tests performed as part of a Radiation Therapy QA program.

Semi-Annual QA		
**Test**	**Analysis Method**	**Guidelines recommandations**
Gradient eddy-currents compensation/cross-term check	Siemens service platform	TG 284 (commissioning)
Gradient sensitivity check	Siemens service platform / Siemens customer platform [1]	TG 284 (monthly)
Body coil tuning check	Siemens service platform	TG 284 (monthly)
RF gain calibration check	Siemens service platform	TG 284 (annual)
Transmitter gain stability check	Siemens service platform	TG 284 (monthly)
Slice position/thickness synthesizer check	Siemens service platform / Siemens customer platform [1]	TG 284 (monthly)
LAP Lasers-on-marks verification (laser location is marked on the walls/ceiling around the room)	Visual	TG 284 (monthly)
Table semi-annual test using Aquarius phantom	Siemens clinical platform	TG 284 (annual)

1. These QA tests are available on the Siemens customer platform with MAGNETOM Sola and Vida scanners on syngo XA31 software and later versions, only. Otherwise, the tests are available on the Siemens service platform.

Supplementary Table 4 : MRI Annual Quality Assurance tests performed as part of a Radiation Therapy QA program.

**Annual QA**		
**Test**	**Analysis Method**	**Guidelines recommandations**
Coil QA (for all other coils not verified monthly, e.g. FlexSmall-4)	Siemens service platform	TG 284 (annual)
B_0 inhomogeneity check using dual-echo 3D GRE using the 25cm Siemens sphere of $NiS0_4$ solution	In-house python code	TG 284 (annual)
3D geometric distortion QA without gradient correction on 3DGRADE phantom (gre_ax_LR only)	Spectronics software	TG 284 (commissioning)
Parallel Imaging SNR verification on 3D MPRAGE for SRS (using ACR phantom)	In-house python code	TG 118<br>(no recommendation)
B_0-field lines verification with Gauss meter incl. 5/200 Gauss	Visual	CPQR (annual)
Mechanical table movement QA with ruler (5, 10, 20, 200, 350mm)	Visual	TG 284 (monthly)
Secondary Systems check (incl. injector, anesthesia)	Visual	AAPM Report 100<br>(no recommendation)
Oxygen sensor check	Visual	AAPM Report 100<br>(no recommendation)
Electrical shutdown check	Siemens Technician	TG 284 (annual)
Table movement during emergency check	Siemens Technician	TG 284 (annual)
Quench button check	Siemens Technician	TG 284 (annual)

Supplementary Table 5 : Sequences with important parameters used in the QA program.

Sequence Name	Flip angle/TR/TE/TE_2	Number of Slices	FOV (frequency×phase×slice)	Aquisition Voxel Resolution	Bandwidth	TSE / EPI factor	Scan time
	°/ms/ms/ms		(mm)	(mm)	Hz/pixel		(min:sec)
FLASH3D_geo	20/6.0/2.16/–	480	500×500×480	0.98×0.98×1.0	330	–/–	24:36
EPI_ghost_geo	90/4860/61/–	34	240×240×170	1.88×1.88×5.0	1260	–/128	0:6
TSE_ghost_geo	150/4860/59/–	34	240×240×170	1.88×1.88×5.0	465	17/–	1:24
EPI_stability	90/3370/40/–	32	260×260×159	4.06×4.06×4.0	1202	–/64	8:30
FLASH3D_B_0	20/22/9.53/19.06	128	280×280×282	2.19×2.19×2.2	540	–/–	9:25

1. These QA tests are available on the Siemens customer platform with MAGNETOM Sola and Vida scanners on syngo XA31 software and later versions, only. Otherwise, the tests are available on the Siemens service platform.

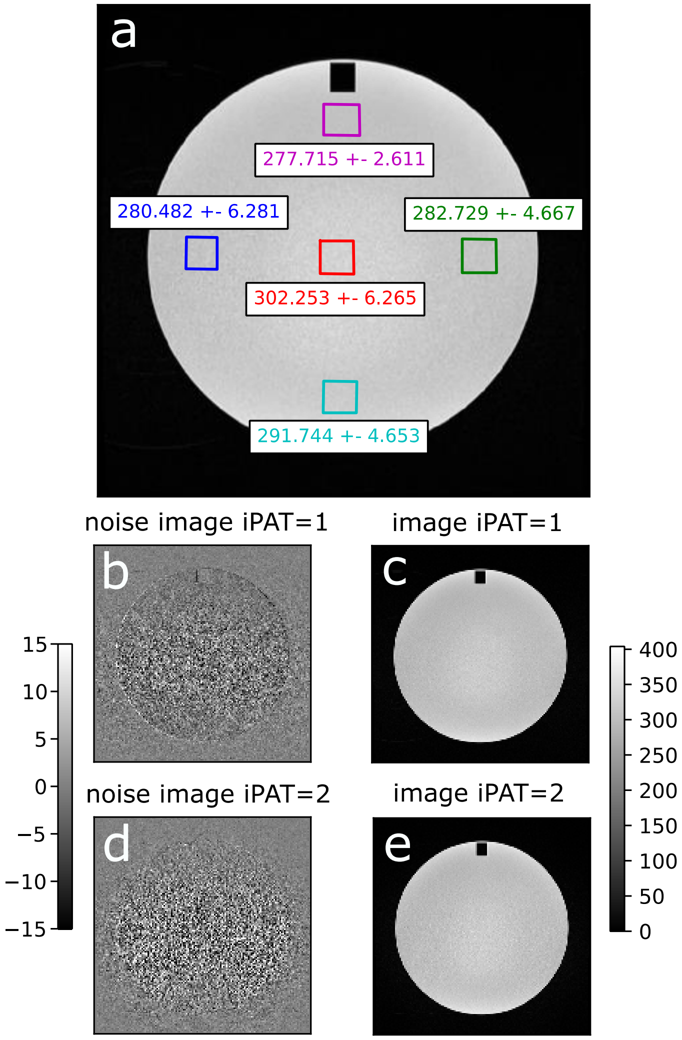

Supplementary Figure 1 : Parallel imaging SNR stability test showing a) ROI regions within the ACR phantom, b) noise image with iPAT = 1, c) image with iPAT = 1, d) noise image with iPAT = 2, e) image with iPAT = 2.

1. These QA tests are available on the Siemens customer platform with MAGNETOM Sola and Vida scanners on syngo XA31 software and later versions, only. Otherwise, the tests are available on the Siemens service platform.

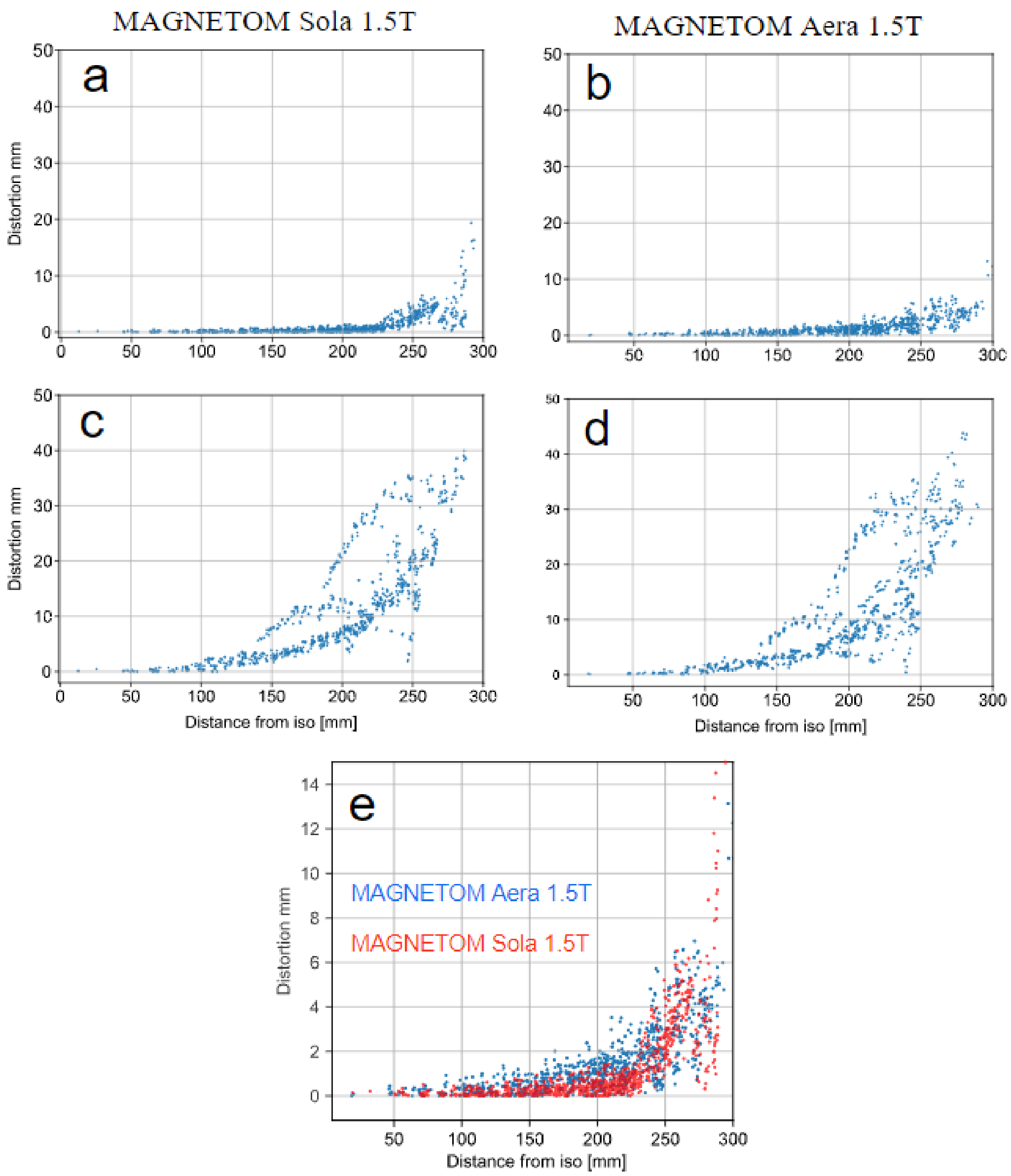

Supplementary Figure 2 : Comparison of Siemens MAGNETOM Sola (a & c) vs MAGNETOM Aera (b & d) distortion vector magnitude plots with (a-b) and without (c-d) gradient non-linearity correction (using the 3D GRADE phantom by Spectronics) for the LR phase-encode direction as a function of control point distance from the MRI isocentre. e) The gradient-corrected control point's distortions of both scanners are superposed on the same axes demonstrating significantly lower mean distortions in the MAGNETOM Sola compared to the MAGNETOM Aera scanner.

1. These QA tests are available on the Siemens customer platform with MAGNETOM Sola and Vida scanners on syngo XA31 software and later versions, only. Otherwise, the tests are available on the Siemens service platform.
